# Supplementary material for: Integrated analysis of the aqueous humor microbiome and lens capsule transcriptome in high myopia cataract: a pilot study
Source: Front Med (Lausanne). 2026 Jun 16;13:1845205. doi: 10.3389/fmed.2026.1845205 (PMC13314463; doi:10.3389/fmed.2026.1845205)
Supplement: Supplementary file 3 [file Data_Sheet_1.zip › 4.Meta_Statistic/C372089/LEfSe/LEfSe_table.cladogram-add_genuslabel.pdf]

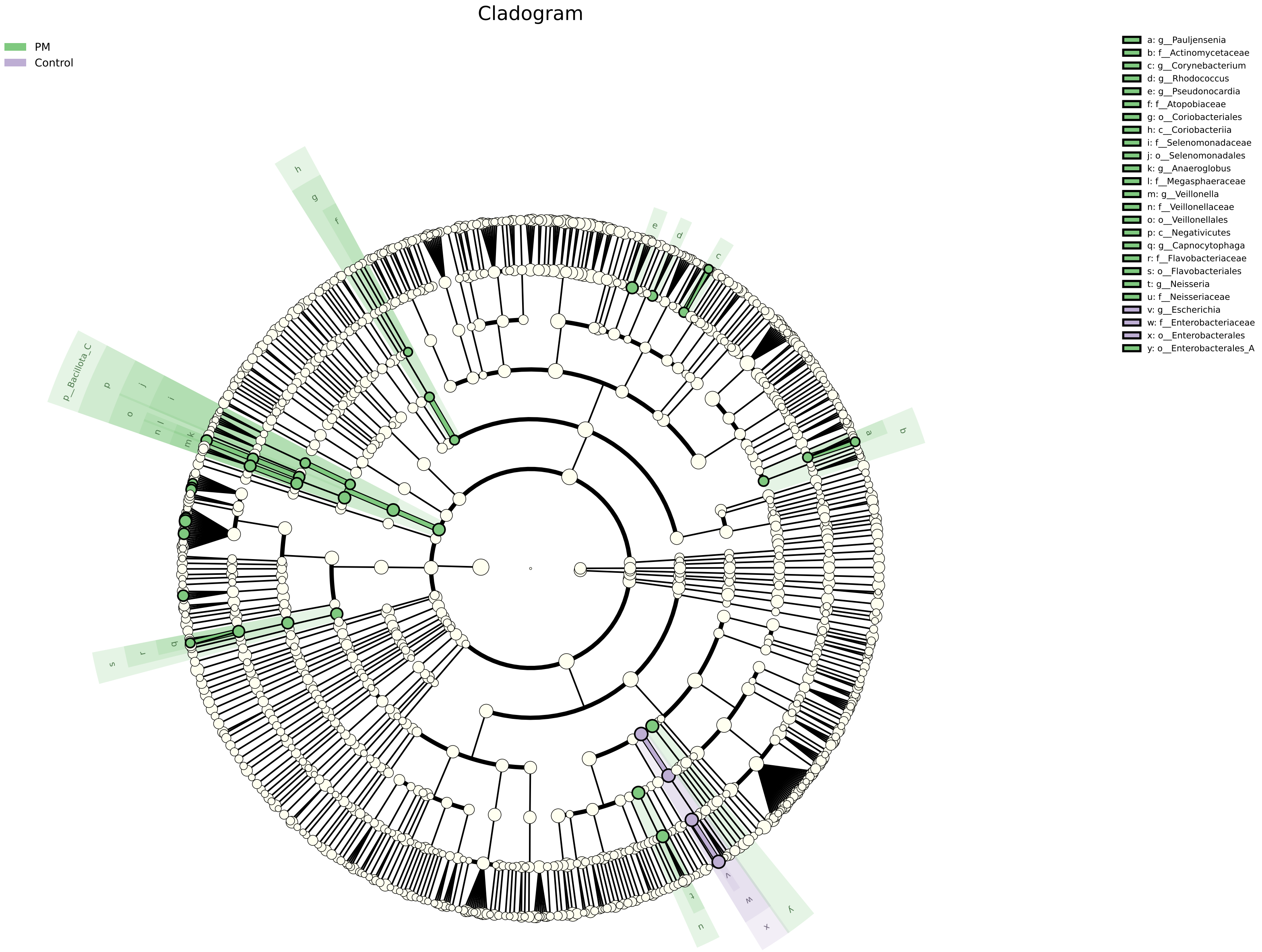
Cladogram
PM
Control
a: g__Pauljensenia
b: f__Actinomycetaceae
c: g__Corynebacterium
d: g__Rhodococcus
e: g__Pseudonocardia
f: f__Atopobiaceae
g: o__Coriobacteriales
h: c__Coriobacteriia
i: f__Selenomonadaceae
j: o__Selenomonadales
k: g__Anaeroglobus
l: f__Megasphaeraceae
m: g__Veillonella
n: f__Veillonellaceae
o: o__Veillonellales
p: c__Negativicutes
q: g__Capnocytophaga
r: f__Flavobacteriaceae
s: o__Flavobacteriales
t: g__Neisseria
u: f__Neisseriaceae
v: g__Escherichia
w: f__Enterobacteriaceae
x: o__Enterobacterales
y: o__Enterobacterales_A
p__Bacillota_C
